# PI3Kδ inhibition prevents IL33, ILC2s and inflammatory eosinophils in persistent airway inflammation

**DOI:** 10.1186/s12865-021-00461-5

**Published:** 2021-12-17

**Authors:** Sorif Uddin, Augustin Amour, David J. Lewis, Chris D. Edwards, Matthew G. Williamson, Simon Hall, Lisa A. Lione, Edith M. Hessel

**Affiliations:** 1 Immunology Research Unit, Respiratory Therapy Area Unit, GSK Medicines Research Centre, GlaxoSmithKline Research and Development Limited, Gunnels Wood Road, Stevenage, Hertfordshire, SG1 2NY UK; 2 In Vivo/In Vitro Translation, GlaxoSmithKline Research and Development Limited, Gunnels Wood Road, Stevenage, Hertfordshire, SG1 2NY UK; 3 Department of Clinical and Pharmaceutical Sciences, School of Life and Medical Sciences, University of Hertfordshire, College Lane, Hatfield, Hertfordshire, AL10 9AB UK; 4 Eligo Bioscience, 29 Rue du Faubourg Saint-Jacques, 75014 Paris, France

**Keywords:** PI3Kδ, IL33, ILC2, Goblet cell metaplasia, Siglec-F^hi^ inflammatory eosinophils

## Abstract

**Background:**

Phosphoinositide-3-kinase-delta (PI3Kδ) inhibition is a promising therapeutic approach for inflammatory conditions due to its role in leucocyte proliferation, migration and activation. However, the effect of PI3Kδ inhibition on group 2 innate lymphoid cells (ILC2s) and inflammatory eosinophils remains unknown. Using a murine model exhibiting persistent airway inflammation we sought to understand the effect of PI3Kδ inhibition, montelukast and anti-IL5 antibody treatment on IL33 expression, group-2-innate lymphoid cells, inflammatory eosinophils, and goblet cell metaplasia.

**Results:**

Mice were sensitised to house dust mite and after allowing inflammation to resolve, were re-challenged with house dust mite to re-initiate airway inflammation. ILC2s were found to persist in the airways following house dust mite sensitisation and after re-challenge their numbers increased further along with accumulation of inflammatory eosinophils. In contrast to montelukast or anti-IL5 antibody treatment, PI3Kδ inhibition ablated IL33 expression and prevented group-2-innate lymphoid cell accumulation. Only PI3Kδ inhibition and IL5 neutralization reduced the infiltration of inflammatory eosinophils. Moreover, PI3Kδ inhibition reduced goblet cell metaplasia.

**Conclusions:**

Hence, we show that PI3Kδ inhibition dampens allergic inflammatory responses by ablating key cell types and cytokines involved in T-helper-2-driven inflammatory responses.

**Supplementary Information:**

The online version contains supplementary material available at 10.1186/s12865-021-00461-5.

## Background

Inflammation is maintained due to presence of specific cells responsible for the rapid production of cytokines. For example, group 2 innate lymphoid (ILC2) cells have been identified as key promoters of eosinophilic persistence in allergic airway inflammation [1]. Inflammatory conditions observed in asthma can be recapitulated in murine models of house dust mite (HDM)-induced persistent pulmonary inflammation [2]. The complex composition of HDM enables interaction with structural and immune cells present in the lung [3, 4]. Due to these inherent properties and the fact that HDM is a clinically important allergen, it is believed the development of murine models utilising HDM as the allergic insult are clinically relevant [5].

Despite much research in the asthma field, inhaled corticosteroids (ICS) have remained a mainstay of anti-inflammatory treatment. However, the symptoms of some patients persist even though high doses of ICS are prescribed [6] and there is a need for non-steroidal or novel approaches in these patient groups. Montelukast, a cysteinyl leukotriene D4 receptor antagonist, is also used as an anti-inflammatory treatment for asthma [7, 8]. However, clinical evidence demonstrates that its effectiveness may be more asthma endotype dependent [9, 10] and indeed montelukast may be more appropriate for atopic paediatric and exercise-induced asthma sub-populations [11]. Antibodies such as Mepolizumab that target IL5, the cytokine responsible for the activation, proliferation, maturation and survival of eosinophils, have been approved for the treatment of eosinophilic diseases including severe refractory eosinophilic asthma [12]. Phosphatidylinositol 3-kinase delta (PI3Kδ) inhibitors have also been developed as potential novel non-steroidal anti-inflammatory agents for asthma and other pulmonary inflammatory disorders [13–16].

The rationale for developing PI3Kδ inhibitors to treat asthma originated from the observation that mice in which the kinase subunit of PI3Kδ was either pharmacologically or genetically inactivated had comparable reduction of airway type 2 cytokines, eosinophilia and airway hyper-responsiveness in preclinical models of ovalbumin-induced allergic airway inflammation [17, 18]. However, those pre-clinical observations have not yet translated into clinical benefits in asthma studies with PI3Kδ inhibitors. For example, the PI3Kδ inhibitor nemiralisib did not significantly reduce FEV1 after 28 days of treatment in adults with uncontrolled asthma. However, the levels of pro-inflammatory cytokines including IL5, and IL13 measured in the sputum of patients treated with nemiralisib were reduced when compared to the placebo group [19]. These observations support the potential PI3Kδ inhibitors as anti-inflammatories and further studies are required in order to define the right patient population and clinical setting. While the effect of PI3Kδ inhibitors is well described on TH2-driven airway inflammation [20], currently no data exists on the impact of PI3Kδ inhibition on group 2 innate lymphoid (ILC2) cells. In order to address this question, we evaluated the effect of PI3Kδ inhibition on IL33 induction, accumulation of ILC2 cells, migration of inflammatory (Siglec-F^hi^) eosinophils and initiation of goblet cell metaplasia. These were compared to the effects of montelukast and anti-IL5 antibody treatment.

In the experiments presented here, mice were sensitised to HDM via the topical route to establish a persistent inflammatory profile in the airways and treated with the orally available selective PI3Kδ inhibitor PI-3065 previously used in syngeneic mouse models of cancer [21]. We found that ILC2 cells remained in the airways after a period of resolution. Re-exposure to HDM at a later time-point caused a rapid cytokine induction, reinitiated pulmonary inflammation and revealed infiltration of an inflammatory sub-population of eosinophil along with enhanced IL33 expression and goblet cell metaplasia. Using this model, we demonstrate that PI3Kδ inhibition, but not leukotriene receptor antagonism nor anti-IL5 treatment, reduces the expression of IL33 along with infiltration of ILC2 cells into the airways. In addition, we observed inhibition of inflammatory (Siglec-F^hi^) eosinophil infiltration and goblet cell metaplasia.

## Results

### 3 weeks of repeated topical HDM sensitisation resulted in persistent pulmonary allergic inflammation and cells involved in the allergic response remained in the lung following a period of resolution

**Fig. 1:**
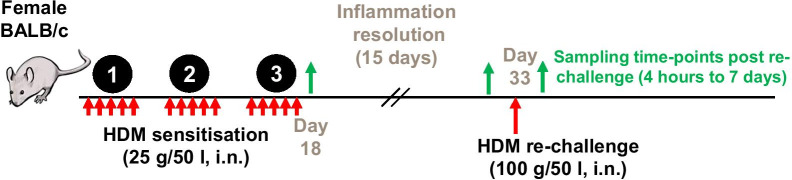
Female BALB/c mice were sensitised with HDM (25 μg/50 μl) via the intra-nasal route, once a day, 5 days a week over a 3 week period (Days 0–18). Inflammation in the airways was allowed to resolve for a period of 2 weeks (Days 19–33). On day 33, respective groups of mice were sacrificed at the end of the resolution period. Other groups of mice were challenged with either saline or re-challenged with HDM (100 µl/50 µl) and relevant groups were sacrificed at various time-points post re-challenge (4 h–7 days) to quantify; cells within the lung tissue and cells and soluble mediators in broncho-alveolar lavage. For studies involving therapeutics, mice were sacrificed at 72 h post re-challenge to quantify myeloid subsets (eosinophils) or lymphoid subsets (ILC2, CD4 positive subsets and B cells). All HDM sensitisation and re-challenges indicated by red arrows. All terminal endpoints are indicated by green arrows

**Fig. 2:**
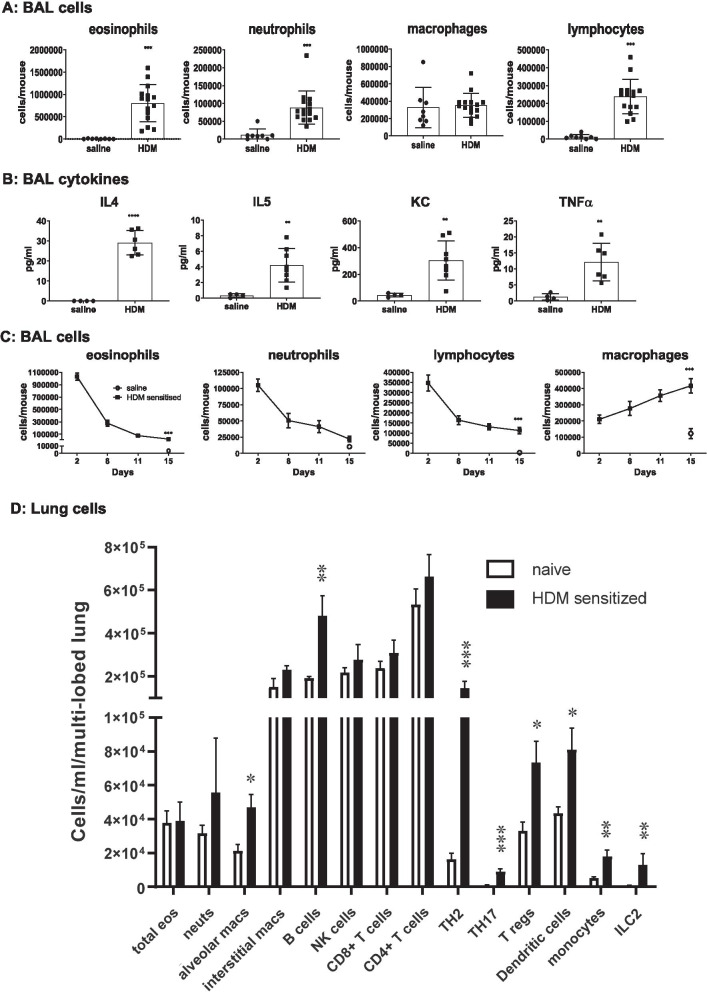
Characterisation of the cell populations present in the airways at the end of the sensitisation or during and after resolution. **A** Three weeks of repeated HDM exposure resulted in a granulocytic and lymphocytic infiltration into bronco-alveolar lavage and also **B** induced an inflammatory mediator response in the airways. **C** This inflammatory profile was allowed to resolve over a 15 day period. At the end of resolution, eosinophils, lymphocytes and macrophages remained in the broncho-alveolar lavage in significantly increased numbers compared to saline sensitized controls. Neutrophil numbers were not different to saline controls, (n = 4–8 mice per group in saline controls and 6–15 in HDM groups). ***p < 0.001 compared to saline controls. **D** Cell populations present in lung tissue at the end of the 15 day resolution period. n = 4 mice per naïve or HDM group. Data analysed using one-way analysis of variance (ANOVA) with Dunnets *p* value adjustment, *p < 0.05, **p < 0.01, ***p < 0.001 compared to naïve unsensitised mice

The challenge and sensitisation protocol used in the current study (Fig. 1) resulted in establishment of an eosinophilic, neutrophilic and lymphocytic infiltration (*p* < 0.001, Fig. 2A) into the airways of mice and induced pro-inflammatory cytokines (*p* < 0.01, Fig. 2B). Cessation of HDM dosing caused resolution of inflammation, except for macrophages, which persisted and increased over time. In addition, lymphocytes and eosinophils were present in significant numbers in the BAL of HDM sensitised mice at the end of the resolution period compared to mice exposed to saline (Fig. 2C). Further investigation of lung tissue cells in HDM sensitised mice at the end of the resolution period revealed a sustained presence of ILC2, TH2, TH17, T-regs, B cells, dendritic cells and monocytes (*p* < 0.05 compared to naïve mice, Fig. 2D).

### Re-challenge to HDM induced rapid cytokine and chemokine production followed by infiltration of myeloid and lymphoid subsets into the lung tissue

**Fig. 3:**
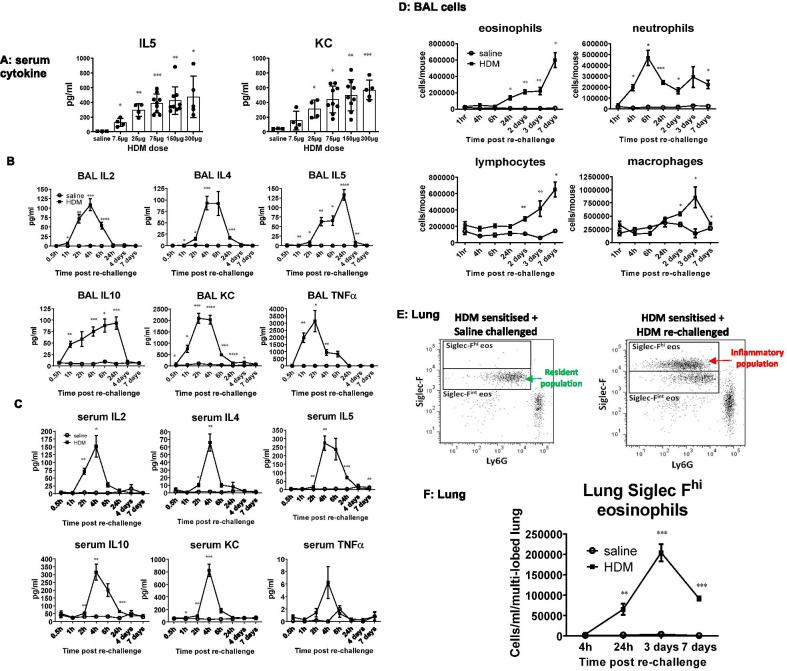
Re-exposure to HDM after resolution on day 33 re-initiated pulmonary inflammation, which was characterised by rapid cytokine production and by leukocyte infiltration. **A** The dose of HDM required to elicit a submaximal (ED80) response in BAL IL5 and KC was 100 µg/50 µl and this dose was used for all subsequent re-challenge experiments (n = 4–11 per dose group). **B** All BAL and **C** serum cytokine responses peaked between 2 and 4 h post HDM re-challenge, with the exception of BAL IL5, which peaked at 24 h. Mice sensitised with HDM but challenged with saline did not exhibit cytokine responses in either BAL or serum, (n = 3–5 per time-point in saline controls and 4–11 mice per time-point for the HDM re-challenged group). **D** After re-challenge with HDM, the earliest leukocyte response was that of neutrophils which peaked at 6 h and remained in higher numbers 7 days post re-challenge compared to saline controls. BAL macrophage numbers peaked at 3 days and returned to saline control levels by day 7. Eosinophils were present in the BAL of HDM re-challenged mice at 24 h, whereas lymphocytes were evident from 2 days post HDM re-challenge (n = 3–5 mice per time-point in saline controls and 8–10 mice per time-point for the HDM re-challenged group). **E** Exemplar dot plot graph demonstrating difference between resident (Siglec-F^int^) eosinophil population and the infiltrating inflammatory eosinophil (Siglec-F^hi^) population. **F** Infiltrating lung inflammatory eosinophils, absent in HDM sensitised and saline challenged controls peaked at day 3 post re-challenge, but were present in numbers from 24 h after re-challenge and were differentiated from resident populations based on Siglec-F expression, (n = 4–10 per time-point for saline controls and 6–17 for HDM re-challenged group). *p < 0.05, **p < 0.01, ***p < 0.001 compared to saline

**Fig. 4:**
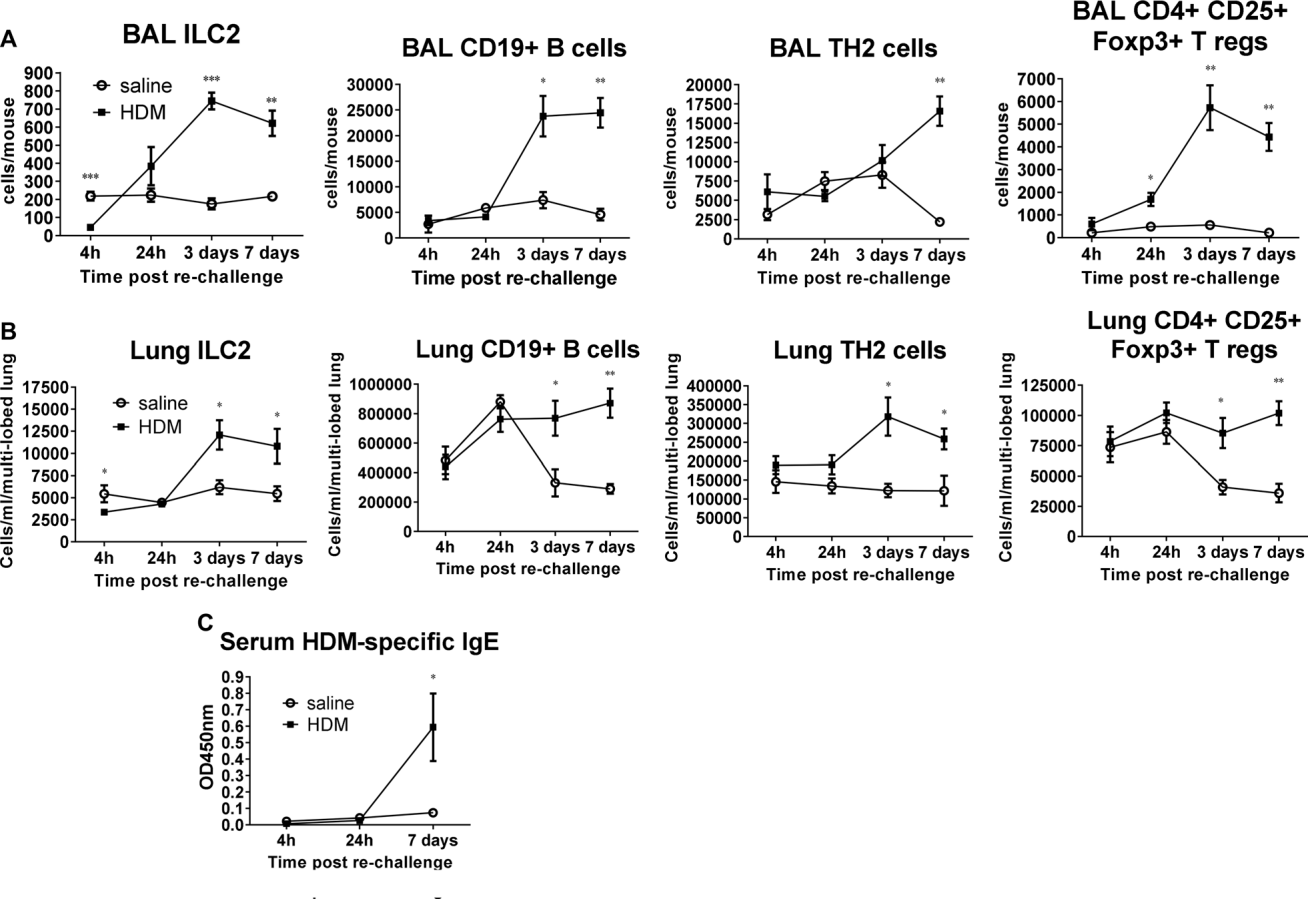
HDM re-challenge initiated a lymphocyte response in the BAL and lungs and HDM specific IgE responses in serum. **A** BAL numbers of ILC2, B and CD4+ T regulatory cells, peaked at 3 days post HDM re-challenge. TH2 cells were only present in numbers at 7 days post HDM re-challenge, (n = 4 per time-point for saline controls and 6 for HDM re-challenged group). **B** Lung lymphocyte responses either peaked at day 3 post HDM re-challenge (ILC2 and TH2 cells) whereas B and T regulatory cell numbers remained constant and did not resolve, (n = 4 per time-point for saline controls and 6 for HDM re-challenged group). **C** HDM-specific IgE was detectable in significant amounts at day 7 in the serum of HDM re-challenged mice coinciding with sustained numbers of CD19^+^ B cells in both BAL and lung (n = 7 in saline groups and 9 in HDM re-challenged groups per time-point). *p < 0.05, **p < 0.01, ***p < 0.001 compared to saline

BAL and serum cytokine responses using 100 µg of HDM re-challenge (Fig. 3A) peaked between 2 and 4 h post HDM re-challenge except for BAL IL5 which peaked at 24 h (Fig. 3B, C). HDM re-challenge (of HDM sensitised mice) evoked a rapid neutrophil response in the BAL, peaking at 6 h. The numbers in the BAL stabilised between 24 h and 7 days post re-challenge but remained above saline challenged levels (Fig. 3D). Macrophage numbers in BAL peaked at 3 days post HDM re-challenge and returned to saline levels by day 7 (Fig. 3D). Eosinophil and lymphocyte numbers in BAL followed a similar time-course of infiltration into BAL post HDM re-challenge, both apparent in significant numbers at 2 days compared to saline controls (*p* < 0.01, Fig. 3D). Infiltrating lung inflammatory eosinophils (Siglec-F^hi^) peaked at day 3 post re-challenge and were present in significant numbers from 24 h after re-challenge. These cells were differentiated from resident populations based on Siglec-F expression (Fig. 3E, F). Lymphocyte subsets in BAL (ILC2, B and CD4^+^Treg cells) peaked at day 3 post HDM re-challenge except for TH2 cells which were present in significant numbers at day 7 (*p* < 0.01 at day 7, Fig. 4A and Additional file 1: Figure E1). In lung, lymphocyte numbers peaked at day 3 post HDM re-challenge (ILC2 and TH2 cells) or persisted in elevated numbers (B and T regulatory cells) compared to saline challenged controls where numbers of these cells continued to resolve (Fig. 4B and Additional file 1: Figure E1). Sustained presence of CD19^+^ B cells in the lung was associated with elevated levels of HDM-specific IgE at day 7 (*p* < 0.05, Fig. 4C).

### Inhibition of PI3Kδ but not antagonism of the leukotriene D4 receptor significantly reduced both cytokine response and infiltration into the airways of ILC2 and inflammatory eosinophils post HDM re-challenge

**Fig. 5:**
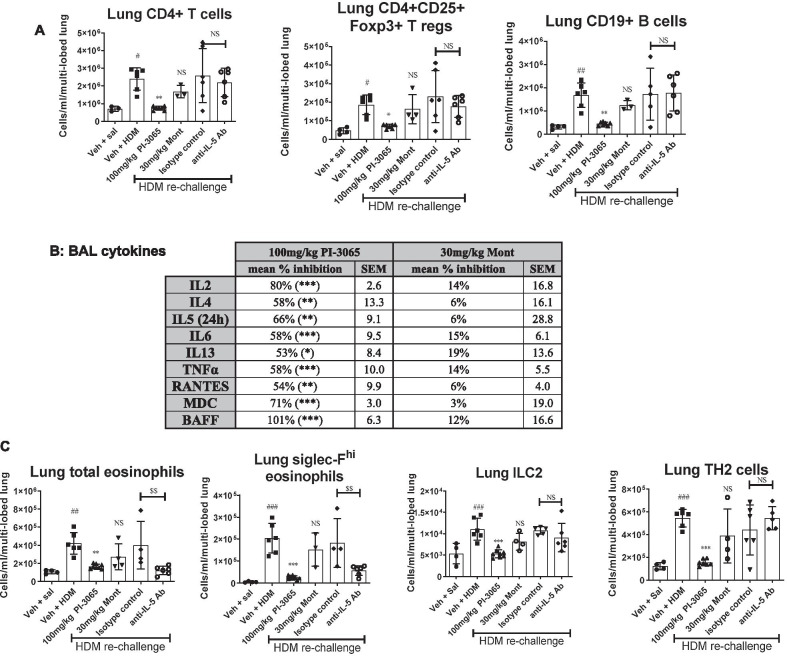
Effect of PI3Kδ inhibition (PI-3065), cysteinyl leukotriene receptor 1 antagonism (montelukast) and anti-IL5 neutralizing antibody on the inflammatory response evoked by HDM re-challenge. All myeloid subsets (eosinophils) were analysed at 24 h post HDM re-challenge, while all lymphoid subsets (ILC2, CD4^+^ subsets and B cells) were analysed at the 3 day time-point. **A** Infiltration of lymphocyte subsets (CD4^+^, T regulatory cells and CD19^+^ B cells) was inhibited by PI-3065 but not montelukast. **B** Cytokines were analysed at 4 h post HDM re-challenge, except for IL5 which was analysed at 24 h post HDM re-challenge. PI-3065 inhibited the induction of cytokines in BAL whereas montelukast was not able to significantly inhibit any of the cytokines investigated. Data expressed as percentage inhibition. **C** PI-3065 and anti-IL5 antibody inhibited the infiltration of eosinophils including the inflammatory subset expressing high levels of Siglec-F into inflamed airways. However, only PI-3065 inhibited the infiltration of ILC2 and TH2 cells, whereas montelukast and anti-IL5 antibody did not significantly inhibit infiltration of these important cell types. (n = 3–6 per group for saline controls and 6–11 for HDM re-challenged groups). ^#^*p* < 0.05, ^##^*p* < 0.01, ^###^*p* < 0.001, compared to vehicle + saline control group. A one-way analysis of variance (ANOVA) with Dunnets *p* value adjustment was used to analyse the data for significance.*p < 0.05, **p < 0.01, ***p < 0.001, compared to vehicle + HDM re-challenged group. ^$$$^*p* < 0.001, compared to isotype control + HDM re-challenged group. Non-significant result is indicated by NS

Inhibition of PI3Kδ using PI-3065 caused reductions in infiltration of CD4^+^ T helper, CD4^+^CD25^+^Foxp3^+^ T regulatory and CD19^+^ B cells into the airways of mice re-challenged with HDM (*p* < 0.001 at 100 mg/kg PI-3065, Fig. 5A). Moreover, treatment with PI-3065 inhibited a range of BAL cytokines that are important in TH2 cell and eosinophil recruitment and antibody class switching in B cells (Fig. 5B). In line with the inhibition of cytokines, PI-3065 treatment resulted in reductions in recruitment of eosinophils into the lung in particular the Siglec-F^hi^ expressing inflammatory eosinophil sub-population (Fig. 5C), without significantly affecting numbers of lung resident eosinophils (Additional file 1: Figure E2). TH2 and ILC2 cell recruitment was also diminished (*p* < 0.001 at 100 mg/kg PI-3065 in both cases, Fig. 5C). Treatment with montelukast did not exhibit significant inhibition in recruitment of any of the inflammatory cell types nor induction of cytokines in the BAL (Fig. 5B). As expected, and previously published, neutralisation of IL5 resulted in significant inhibition of eosinophil migration, including inflammatory eosinophils (Fig. 5C). However, anti-IL5 antibody treatment did not inhibit either TH2 or ILC2 cells (*p* > 0.05 when compared to IgG1 isotype control, Fig. 5C).

### PI3Kδ inhibition but not montelukast or anti-IL5 antibody treatment resulted in reduced IL33 expression and improvement of goblet cell metaplasia

**Fig. 6:**
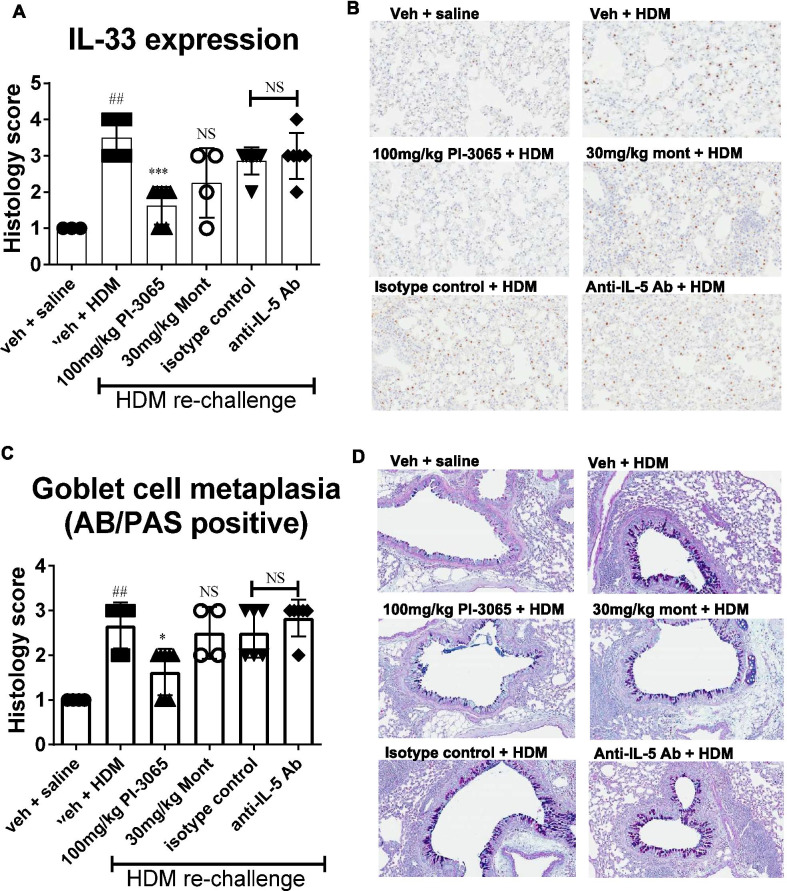
Effect of treatment on IL33 expression and goblet cell metaplasia assessed by histological analysis. **A** Interleukin-33 expression as assessed by immuno-histochemistry and blinded semi-quantitative scoring, 24 h after re-challenge with HDM. IL33 expression was localised to Type II pneumocytes identified by location and morphology. HDM re-challenge resulted in significant expression of IL33 in Type II pneumocytes compared to saline challenged controls. Treatment with PI-3065 significantly ablated IL33 expression in Type II pneumocytes, whereas montelukast or anti-IL5 antibody treatment were ineffective at inhibiting IL33 expression. **B** Representative histological images (× 20 magnification) of IL33 expression in each treatment group. **C** Identification of goblet cell metaplasia was carried out by Alcian Blue/Periodic Acid Schiff tinctorial staining at the 72 h time-point post HDM re-challenge. **D** Representative histological images of AB/PAS positive goblet cells in each treatment group. Treatment with PI-3065, but not anti-IL5 antibody or montelukast treatment, was able to reduce goblet cell metaplasia in the airways. Data in **A** and **C** is presented as median with range. Data resulted from blinded immuno-histochemistry scoring and were analysed for significance using Kruskal–Wallis test with Dunn’s multiple comparison post-hoc test. (n = 3 per group for saline controls and 4–8 for HDM re-challenged groups) ^##^*p* < 0.01, compared to vehicle + saline control group. ***p < 0.001, compared to vehicle + HDM re-challenged group

In the HDM re-challenge group, IL33 staining was found in cells located in alveoli with a morphology indicative of type 2 pneumocytes. However, not all type 2 pneumocytes were positive for IL33. HDM re-challenge resulted in significant increase in both the number of positive cells and the intensity of their immunoreactivity (*p* < 0.01 compared to saline controls, Fig. 6A, B). PI3Kδ inhibition, but not montelukast or anti-IL5 antibody treatment caused a statistically significant decrease in IL33 immunoreactivity (*p* < 0.001, Fig. 6A, B). Alcian Blue/ Periodic Acid Schiff (AB/PAS) staining showed a statistically significant increase in the number of goblet cells in the proximal bronchioles of HDM re-challenged mice compared to saline controls (*p* < 0.01, Fig. 6C, D). PI3Kδ inhibition, but not Montelukast or anti-IL5 antibody treatment resulted in a significant reduction in the number of goblet cells present in the airways (*p* < 0.05, Fig. 6C, D).

## Discussion

**Fig. 7:**
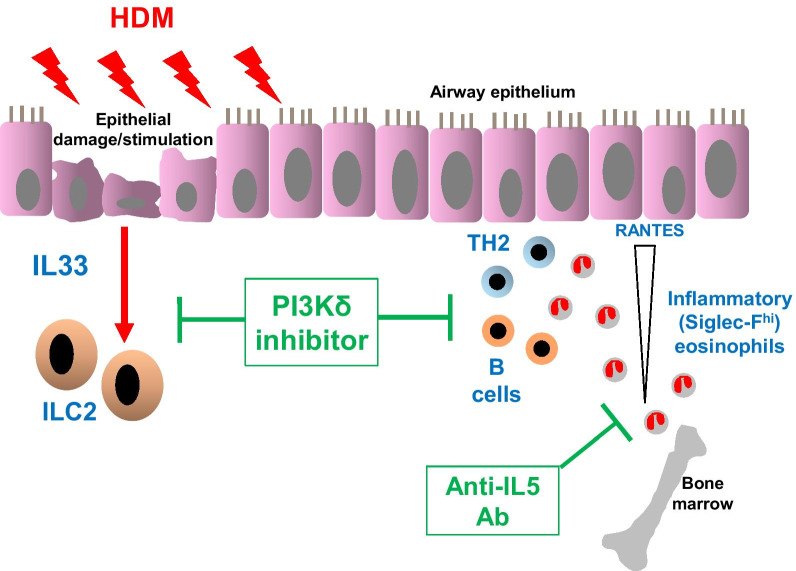
Schematic representation of the key inflammatory pathways involved in allergic pulmonary inflammation and their ablation by PI3Kδ inhibition and anti-IL5 antibody treatment

We demonstrate that PI3Kδ inhibition, but not leukotriene D4 receptor antagonism or IL5 neutralisation, inhibits the expression of IL33 and accumulation of ILC2 cells in the airways (Fig. 7). Migration of inflammatory eosinophils was attenuated by both PI3Kδ inhibition and IL5 neutralisation but not by montelukast. Moreover, airway goblet cell metaplasia was ablated by PI3Kδ inhibition but not by montelukast or anti-IL5 antibody treatment.

Modelling the pulmonary allergic inflammatory process in animals can be challenging and appropriate animal models reflecting all aspects of human disease do not exist [5, 22]. To assess the impact of novel therapeutic approaches and to benchmark established therapies, we sought to develop a murine system which modelled the processes of airway sensitisation, resolution and re-exposure to allergen as observed in asthma patients using a clinically relevant allergen.

We found that sensitisation by repeated airway exposure using HDM, results in persistent pulmonary allergic inflammation without the need for systemic adjuvants, as has been reported previously by Johnson and colleagues [2]. In addition, the mouse model reported in this paper employed a period where there was no HDM exposure to mimic resolution of the inflammatory portion of the airway sensitisation response. The profile of inflammatory cell types, which included ILC2 cells, in the airways of mice at the end of the resolution period was found to be similar to that observed in eosinophilic asthma patients [23, 24].

ILC2 cells are critical in rapidly mounting and maintaining TH2 type cellular responses to the airways and promote migration of dendritic cells to local draining lymph nodes [25]. They are found in increasing numbers in the airways of severe asthmatics [26] and upon activation they rapidly produce a range of TH2 cytokines such as IL2, IL4, IL5 and IL13 [27], even when high doses of oral corticosteroid are used [1]. In addition, ILC2 cells have been identified as key biomarkers of eosinophilic airway inflammation in asthma patients [28] and as cells that are responsible for exacerbations [29]. ILC2 cells are activated in the presence of epithelial damage-induced cytokines such as IL33 [27, 30–32], the expression of which is increased in asthmatics [32].

In the current mouse model, a single allergen re-challenge at the end of the resolution period resulted in a re-induction of TH2 cytokines, enhanced expression of IL33 in the tissue, amplified goblet cell metaplasia and increased accumulation of myeloid and lymphoid cells, including ILC2 cells, in the airways. Eosinophils in the tissue were sub-divided into two populations based on the expression of Sialic acid-binding immunoglobulin-type lectin-F (Siglec-F). A stable resident population of eosinophil that expressed intermediate levels of Siglec-F (Siglec-F^int^) and an infiltrating population, apparent only after allergen re-challenge expressing high levels of Siglec-F (Siglec-F^hi^). Mesnil and colleagues in a murine model also using HDM [33, 34] showed that Siglec-F^int^ eosinophils were important for immune regulation and homeostasis and were differentiated from inflammatory eosinophils [33].

Using the current mouse model, we chose to study the effect of therapeutics on a pre-inflamed background prior to allergen re-challenge as would occur in allergic asthma patients. Given the position of ILC2 cells as initiators of the allergic inflammatory cascade, we sought to determine if our model could be used to determine the effect of current and future therapies on their function. Of the standard non-steroidal therapies used for asthma management, we did not find any reports documenting the direct inhibitory effect of anti-IL5 antibody treatment on the activation or accumulation of ILC2 cells in the airways. Our data clearly show that this treatment had no effect on the increase in ILC2 or TH2 cell numbers in the airways, hence we conclude that migration of both these cell types into inflamed airways was not dependent on IL5. However, anti-IL5 antibody treatment inhibited the migration of the Siglec-F^hi^ inflammatory eosinophils, a finding that is consistent with their reported reliance on IL5 for migration to the airways [33].

Montelukast, a cysteinyl leukotriene D4 receptor antagonist has been shown to inhibit IL4 production in isolated human ILC2 cells [35]. However, this is in contrast to our own findings, where in a complex in vivo setting, montelukast failed to inhibit not only IL4 but also other cytokines and chemokines involved in the allergic response. Moreover, montelukast did not inhibit ILC2 infiltration into inflamed airways, hence we conclude that migration of ILC2 cells to the airways in the current mouse model was not dependent on eicosanoids such as leukotriene D4. In addition, we did not observe any effect of montelukast dosing on the accumulation of Siglec-F^hi^ inflammatory eosinophils in the airways.

Our data demonstrates that the PI3Kδ inhibitor PI-3065 reduces the expression of IL2 and IL33, key mediators of ILC2 cell proliferation and activation [36, 37]. As a consequence, we observed a profound inhibition of ILC2 accumulation into inflamed airways with PI-3065 treatment. Overall, our data suggest a critical function of PI3Kδ which to our knowledge has not been reported. This function is in addition to the known roles of PI3Kδ in allergic asthma such as; leucocyte migration into inflamed tissues [38–40], release of asthma relevant cytokines (IL4 and IL5) [18] and chemokines (RANTES and eotaxin) [17]. Moreover, PI-3065 treatment reduced airway goblet cell metaplasia which was concomitant with reduction in IL13 levels, a cytokine that induces goblet cell metaplasia in the airways [41, 42]. We also report for the first time that PI3Kδ inhibition attenuated the migration of Siglec-F^hi^ inflammatory eosinophils into the airways post allergen re-challenge without effecting the lung resident and regulatory Siglec-F^int^ eosinophil population. Furthermore, PI-3065 reduced levels of RANTES (CCL5), another important mediator for the haematopoiesis, survival and chemotaxis of eosinophils to asthmatic airways [43].

In these studies, we have developed a murine model incorporating sensitisation, resolution and allergen re-challenge which allowed us to uncover the persistence of ILC2 cells in the airways of mice. Airway accumulation of this cell type was found to be PI3Kδ dependent. Re-challenge to allergen after a period of inflammation resolution, uncovered an inflammatory population of eosinophils, the migration of which we found to be dependent on PI3Kδ. Key cytokines that activate ILC2 cells (IL33) or induce goblet metaplasia (IL13), were ablated by PI3Kδ inhibition and cytokines resulting from ILC2 activation were also dampened. To date PI3Kδ inhibitors tested in asthmatics have not met the primary endpoint of improving lung function in their clinical trials. This indicates a lack of translatability of our model with asthma, which is a heterogeneous disease resulting from a combination of multiple factors beyond allergens. Of notes, the levels of the type 2 pro-inflammatory cytokines including IL5, and IL13 measured in the sputum of patients treated with nemiralisib were reduced when compared to those treated with placebo [19]. This agrees with a critical role for PI3Kd in type 2 inflammatory cells including ILC2s. Our data combined underscores the need to define the right allergic patient population and clinical setting in which ILC2s play a critical pathological role [44].

## Conclusions

In this study, we develop a murine system which models the processes of airway sensitisation, resolution and re-exposure to allergen. In the model, we uncover the persistence of ILC2 cells in the airways of mice, which we show to be sensitive to PI3Kδ inhibition. Our results point towards a patient stratification approach based on elevated numbers of ILC2 cells in the targeted tissue. Such an innovative approach may lead to improved clinical outcomes for PI3Kδ inhibitors in refractory allergic and inflammatory conditions.

## Methods

### Animals

Female BALB/c mice, aged 6–8 weeks, weighing approximately 20 g were purchased from Charles River UK Ltd, acclimatised for seven days and randomly assigned to control or treatment groups. All animal related protocols were reviewed and approved by the Animal Welfare Ethical Review Body (AWERB) of GSK. All animal studies were ethically carried out in accordance with relevant guidelines and regulations including the Animals (Scientific Procedures) Act 1986 and the GSK Policy on the Care, Welfare and Treatment of Animals and in compliance with the ARRIVE guidelines. Mice were given food and water ad libitum. For all investigations, mice were sacrificed using an intra-peritoneal overdose of sodium pentobarbitone (Dolethal, 200 mg/ml, Vetoquinol, UK).

### HDM sensitisation, inflammation resolution and re-challenge protocols

For all intranasal (i.n.) administrations, mice were anaesthetised using 2% isofluorane in oxygen. HDM sensitisation was carried out by i.n. dosing once a day for 5 days a week over a 3 week period (Days 0–18) with either sterile saline or 25 μg HDM extract (Greer Laboratories, Lenoir, NC, USA) in 50 μl sterile saline. Pulmonary inflammation was then allowed to resolve until day 33. Inflammation was then re-initiated by an i.n. re-challenge of HDM using 100 µg of HDM extract (100 μg) in 50 μl saline. Respective groups of mice were sacrificed at the end of the sensitisation period (day 33) and following re-challenge at pre-determined time-points (0.5 h–7 days) to ascertain the cellular and cytokine composition in both BAL and lung tissue.

### Compound and antibody dosing protocols

Mice were dosed with the PI3Kδ inhibitor PI-3065, montelukast or vehicle (sterile distilled water containing 0.5% Hydroxypropyl methylcellulose (Sigma-Aldrich, UK), 0.2% Tween-80 (Sigma-Aldrich, UK)) via oral gavage in a total volume of 0.2 ml. This occurred once daily for 7 days starting on day 26 (7 days prior to re-challenge) and ending on the day of HDM re-challenge. Anti-IL5 neutralizing antibody (100 μg/mouse, Clone number: TRFK-5, BD Biosciences, UK) or Rat IgG1 (Clone number: R3-34, BD Biosciences, UK) were dosed intra-peritoneally in 0.2 ml sterile saline, 1 h prior to HDM re-challenge. Please refer to the Additional file 1: Figure E3 for the PK profiles of both PI-3065 and montelukast.

### Sample collection, processing procedures and analysis/quantification of cell composition

Please refer to Additional file 1 for full details of sample/tissue (blood, BAL and lung) collection and preparation for cytokine. The panels of flow cytometry antibodies used for the cell composition analysis are described in the Additional file 1 as such: myeloid cell panel (Additional file 1: table S1), lymphocyte panel (Additional file 1: table S2), T helper cell panel (Additional file 1: table S3) and innate lymphoid type 2 cells (Additional file 1: table S4).

### Analysis of cytokines and IgE

Analysis of cytokines in serum and BAL supernatants was carried out as per manufacturer’s instructions using mouse Magnetic Luminex® assay kits (R&D Systems, UK) and mouse V-Plex, Pro-inflammatory Panel 1 kits (Meso Scale Discovery®, US). Serum IgE was analysed using ELISA (please refer to Additional file 1 for more details).

### Histological analysis

Please refer to Additional file 1 for full details of tinctorial (AB/PAS) and immunohistochemistry methods for detection and quantification of goblet cell metaplasia and IL33 expression in lung tissue.

### Statistical analysis

Except where stated, data in all figures is presented as arithmetic means ± standard error of the mean (SEM). Numbers of mice per group were ascertained from institutional experience gained during the development of the HDM re-challenge model and are indicated in the figure legends. Only histological assessment was performed blinded and no data points were excluded from any experiments. Except where stated, statistical analysis was performed using GraphPad Prism 5 and where necessary data were log transformed and a one-way analysis of variance (ANOVA) with Dunnets *p* value adjustment was used to analyse the data for significance. Statistical significances of HDM challenge or drug treatment effects were performed by comparing HDM challenged mice or drug treatment groups to their respective corresponding naïve mice counterparts or saline control groups with a two-tailed Student’s t test using Excel version 2002 (Microsoft Corporation, Redmond, Washington). *p* values of less than or equal to 0.05 were considered significant.

## Supplementary Information

**Additional file 1.** Online methods supplement.

## Data Availability

The datasets generated and/or analysed during the current study are not publicly available due to privacy restrictions but are available from the corresponding author on reasonable request.

## Abbreviations

PI3Kδ: Phosphoinositide-3-kinase-delta
IL2: Interleukin-2
IL4: Interleukin-4
IL5: Interleukin-5
IL10: Interleukin-10
IL13: Interleukin-13
IL33: Interleukin-33
KC: Keratinocyte chemoattractant protein
TNFα: Tumour Necrosis factor alpha
ILC2: Group 2 innate lymphoid cell
IgE: Immunoglobulin E
TH2: T helper type 2 cells
HDM: House dust mite
BAL: Broncho-alveolar lavage
RANTES: Regulated on Activation, Normal T Cell Expressed and Secreted
