# Supplementary material for: PI3Kδ inhibition prevents IL33, ILC2s and inflammatory eosinophils in persistent airway inflammation
Source: BMC Immunol. 2021 Dec 17;22:78. doi: 10.1186/s12865-021-00461-5 (PMC8684271; doi:10.1186/s12865-021-00461-5)
Supplement: Supplementary file 1 — Additional file 1. Online methods supplement. [file 12865_2021_461_MOESM1_ESM.docx]

Online method supplement:

**PI3Kδ inhibition prevents IL33, ILC2s and inflammatory eosinophils in persistent airway inflammation**

Sorif Uddin; Augustin Amour; David Lewis; Chris D Edwards; Matthew G Williamson; Simon Hall; Lisa Lione and Edith M Hessel

**Sample collection and processing procedures**

***Sample collection:***

*Circulating blood sample (for PK analysis)*

Live circulating blood samples were collected from mice at terminal time-points from a tail vein after placing mice into a warm (30^o^C) environment for 10 minutes. For PK analysis, 10μl of blood was placed into the same volume of distilled sterile water and frozen on dry ice and stored at -80^o^C until later analysis.

Mice were then sacrificed using an intra-peritoneal overdose of sodium pentobarbitone (200mg/ml, Vetoquinol, UK) and all sample/tissue collection procedures were carried out in mice after confirmation of death by severance of the spinal column without damaging the trachea.

*Collection of blood for serum*

Blood samples were obtained from severed jugular veins and allowed to clot before processing to obtain serum.

*Broncho-Alveolar Lavage (BAL)*

BAL was performed by instillation, via a tracheal cannula, of 1ml Dulbeccos-phosphate buffered saline (D-PBS, without magnesium and calcium, Gibco UK), containing 10mM EDTA (Sigma-Aldrich, UK), 0.1% BSA (Sigma-Aldrich, UK) and Complete-EDTA free protease inhibitor cocktail (Roche, UK), followed by gentle aspiration. The process was repeated once more using the same aspirated BAL fluid and the final volume recorded.

*Lungs*

Lungs were removed from the thoracic cavity and split into multi-lobed (right) and single lobed (left) lungs. The multi-lobed lung was placed in D-PBS and was used for flow cytometric analysis of cell populations. Where necessary, the single-lobed lung was frozen on dry ice and stored at -80^o^C for later PK analysis.

***Sample processing:***

*Serum*

Clotted blood samples were centrifuged at 7000g for 3 minutes to obtain serum which was then stored at -80^o^C.

*BAL*

Samples of BAL were centrifuged at 370g for 5 minutes after which supernatants were removed and stored at -80^o^C for later cytokine analysis. Cell pellets were resuspended in the original BAL volume using D-PBS containing 1% BSA.

*Lungs*

Multi-lobed lungs were transferred to GentleMACS^TM^ C-Tubes (Miltenyi Biotech, UK) and the lungs were processed to single cell suspensions according to manufacturer’s instructions using the GentleMACS^TM^ Dissociator (Miltenyi Biotech, UK) and mouse lung dissociation kits (Miltenyi Biotech, UK).

**IgE ELISA:**

*Total and HDM-specific IgE:*

Total IgE response was assessed by ELISA. 96-well plates (Maxisorp; Nunc, UK) were coated and incubated overnight at 4^o^C with 50µl of a 1:250 diluted (in PBS) anti-IgE antibody (BD Biosciences, UK, clone: R35-72). For HDM-specific IgE, wells were coated with 50µl of a 1mg/ml solution of HDM extract and incubated as per total IgE plates. From this point onwards, both total and HDM-specific plates were treated the same.

Plates were then washed with PBS containing 0.05% Tween-20 (wash buffer) after which plates were blocked for 1 hour using 100µl/well of PBS containing 4% BSA (Sigma-Aldrich, UK). A standard calibration range of 100 – 1.5ng/ml purified mouse IgE (BD, Biosciences) was prepared using PBS containing 4% BSA. After removing blocking reagent and without washing, 50µl of 1:2000 for total IgE and 1:500 for HDM-specific IgE diluted serum samples or standards were incubated overnight at 4^o^C. Plates were then washed using wash buffer and then incubated for 1 hour at 4^o^C using 50µl of a 1:250 diluted (in PBS containing 4% BSA and 0.05% Tween-20 (assay buffer)) solution of biotinylated anti-IgE antibody (BD Biosciences, clone: R35-118). After washing the plates in wash buffer, 50µl of a 1:4000 diluted (in assay buffer) solution of streptavidin-horse radish peroxidase (Amersham Bioscience, UK) was added to each well and incubated for 30 minutes at room temperature. After washing plates using wash buffer, 100µl of 3, 3`, 5, 5`-tetramethyl benzidine Liquid substrate system (TMB, Sigma-Aldrich, UK) was added to each well for 5-10 minutes, or until the lowest standard had developed colour. The reaction was then stopped using 100µl of 0.25M Sulphuric acid. Optical densities were read at 450nm. Concentrations of total IgE were calculated from the standard curve using Softmax Pro software (Molecular devices, Sunnyvale, California, USA). Raw optical density values at 450nm were plotted for HDM-specific IgE.

**Analysis and quantification of cell composition:**

*Sysmex XT vet 2000i analysis:*

All tissue samples containing single cell suspensions were analysed using an XT vet 2000i flow cytometer (Sysmex, UK) to obtain total and differential (eosinophil, neutrophil, lymphocyte and macrophage) cell counts. Species and tissue specific gating algorithms developed and characterised by Sysmex, UK were used to quantify cell populations.

*FACSCantoII analysis:*

For analysis of lymphocyte and myeloid subsets, a surface and intra-cellular marker staining protocol was used and samples were run on a FACSCantoII machine (Becton Dickinson, UK) running FACSDiva software version 8. Data analysis was performed using FlowJo version 7.6.5 (FlowJo LLC, US).

The staining procedure was as follows (for antibodies, clone number, suppliers, fluorochrome conjugates and dilutions please refer to supplementary tables):

Red blood cells of single cell suspensions containing 1x10^6^ cells were lysed using a 1/10 dilution in sterile distilled water of IO Test 3 solution (Beckman Coulter, UK) for 5 minutes at room temperature. The lysing solution was then washed twice by centrifugation at 370g for 5 minutes at 4^o^C. Supernatants were discarded and cell pellets were resuspended in 20μl Fc-Block^TM^ to block non-specific Fcγ binding sites for 10 minutes at 4^o^C. Without washing off Fc-Block^TM^, cells were stained with 50μl surface marker antibody cocktails for 10 minutes at 4^o^C in the dark after which cells were washed twice (as before) to remove any unbound antibodies. Cell pellets were then resuspended in 200μl of FACS buffer (D-PBS containing 1% Foetal Calf Serum). Data was then acquired on a calibrated FACSCantoII flow cytometer (Becton Dickinson, UK) using fluorochrome compensated protocols. For protocols requiring intra-cellular staining, cell pellets were resuspended in 100μl of Fix/Perm buffer and incubated for 30 minutes at 4^o^C or left overnight. Cells were then washed twice (as before) and cell pellets were resuspended in 50μl of intra-cellular staining solution for 1 hour at 4^o^C in the dark. Cells were then washed twice (as before) and pellets resuspended in 200μl of FACS buffer. Data was then acquired on fluorochrome compensated protocols on a calibrated FACSCantoII machine.

Supplementary table 1:

*Myeloid cell panel (eosinophil, neutrophil, alveolar/interstitial macrophages, dendritic cells and monocytes):*

| **Marker** | **Fluorochrome** | **Dilution** | **Clone #** | **Supplier** |
| --- | --- | --- | --- | --- |
| CD45 | V500 | 1:200 | 30-F11 | Becton Dickinson, UK |
| CD11b | FITC | 1:200 | M1/70 | Biolegend, UK |
| CD64 | PE-Cy7 | 1:200 | X54-5/7.1 | Biolegend, UK |
| CD24 | APC-Cy7 | 1:200 | M1/69 | eBioscience, US |
| MHCII | PerCp-Cy5.5 | 1:200 | M5/114.15-2* | Biolegend 107626 |
| CD11c | APC | 1:400 | N418 | Biolegend, UK |
| Ly6-G | BV421 | 1:400 | 1A8 | Biolegend, UK |
| Siglec-F | PE | 1:50 | ES22-10DB | Miltenyi biotech, UK |

Gating strategy was as described in Misharin, Morales-Nebreda, Mutlu *et al*, 2013. Gating of intact and single cells (forward scatter Height versus Area) was based on forward and side scatter characteristics. Leucocyte populations were identified as CD45^+^ in the singlet gate. Cell subsets were then quantified based on expression of cell surface markers as follows:

Alveolar macrophages: CD11c^hi^CD11b^int^CD64^+^CD24^int^

Interstitial Macrophages: CD11c^+^CD11b^+^CD24^-^MHCII^+^CD64^hi^

Dendritic cells: CD11c^+^MHCII^+^

Residential Eosinophils: CD11c^-^CD11b^+^MCHII^lo^CD24^+^Siglec-F^int^

Inflammatory eosinophils: CD11c^-^CD11b^+^MHCII^lo^CD24^+^Siglec-F^hi^

Neutrophils: CD11c^-^CD11b^+^MHCII^lo^CD24^+^Ly6-G^+^

Supplementary table 2:

*Lymphocyte panel (CD4, CD8, CD19, NK cell and T reg):*

| **Marker** | **Fluorochrome** | **Dilution** | **Clone #** | **Supplier** |
| --- | --- | --- | --- | --- |
| Extracellular Staining |  |  |  |  |
| CD45 | Viogreen | 1:30 | 30-F11 | Miltenyi Biotech, UK |
| CD4 | APC Vio-770 | 1:30 | GK1.5 | Miltenyi Biotech, UK |
| TCRβ | Vioblue | 1:30 | REA318 | Miltenyi Biotech, UK |
| CD19 | Per-Cp-Vio-700 | 1:30 | 6D5 | Miltenyi Biotech, UK |
| CD25 | Pe-Vio-770 | 1:30 | 7D4 | Miltenyi Biotech, UK |
| CD8a | APC | 1:200 | 53-6.7 | Biolegend, UK |
| CD49b | FITC | 1:200 | DX5 | Biolegend, UK |
| Intracellular Staining |  |  |  |  |
| FOXP3 | PE | 1:50 | FJK-16s | eBioscience, US |

Lymphocyte sub-populations were identified as CD45^+^ in the singlet gate. Cell subsets were then quantified based on expression of cell surface markers as follows:

CD4 cells: TCRβ^+^CD4^+^

CD8 cells: TCRβ^+^CD8^+^

B cells: CD45^+^CD19^+^

NK Cells: CD45^+^CD49b^+^

T regulatory cells: TCRβ^+^CD4^+^CD25^+^Foxp3^+^

Supplementary table 3:

*T helper cell panel (TH1, TH2, TH17):*

| **Marker** | **Fluorochrome** | **Dilution** | **Clone #** | **Supplier** |
| --- | --- | --- | --- | --- |
| CD45 | V500 | 1:200 | 30-F11 | BD Biosciences, UK |
| CD4 | APC ALEXA-750 | 1:200 | RM4-5 | Invitrogen, UK |
| TCRβ | BV421 | 1:200 | H57-597 | BD Biosciences, UK |
| CD44 | PE-Cy7 | 1:200 | IM7 | BD Biosciences, UK |
| CD196 (CCR6) | ALEXA-647 | 1:200 | 140706 | BD Biosciences, UK |
| CD183 (CXCR3) | PE | 1:200 | CXCR3-173 | BD Biosciences, UK |
| CD62L | FITC | 1:200 | MEL-14 | BD Biosciences, UK |

Lymphocyte sub-populations were identified as CD45^+^ in the singlet gate. Cell subsets were then quantified based on expression of cell surface markers as follows:

TH1 cells: TCRβ^+^CD4^+^CD44^+^CD62L^-^CD183^+^

TH2 cells: TCRβ^+^CD4^+^CD44^+^CD62L^-^CD183^-^CD196^-^

TH17 cells: TCRβ^+^CD4^+^CD44^+^CD62L^-^CD196^+^

Supplementary table 4:

*Innate lymphoid type 2 cells:*

| **Marker** | **Fluorochrome** | **Dilution** | **Clone #** | **Supplier** |
| --- | --- | --- | --- | --- |
| 7AAD | PerCP-Cy5.5 | 5ul per sample |  | BD Biosciences, UK |
| CD5 | PE-Cy7 | 1:400 | 53-7.3 | Biolegend, UK |
| CD3 | PE-Cy7 | 1:400 | 17-A2 | Biolegend, UK |
| CD49b | PE-Cy7 | 1:400 | DX-5 | Biolegend, UK |
| CD27 | PE-Cy7 | 1:400 | LG-3A10 | Biolegend, UK |
| TCRβ | PE-Cy7 | 1:400 | H57-597 | Biolegend, UK |
| CD11b | APC-Cy7 | 1:400 | M1/70 | Biolegend, UK |
| CD11c | APC-Cy7 | 1:400 | N418 | Biolegend, UK |
| B220 | APC-Cy7 | 1:400 | RA3-6B2 | Biolegend, UK |
| T1/ST2 | FITC | 1:200 | DJ8 | MD Bioproducts, US |
| CD25 | PE | 1:200 | PC61 | Biolegend, UK |
| CD90.2 | V500 | 1:200 | 53-2.1 | BD Biosciences |
| CD127 | BV421 | 1:200 | A7R34 | Biolegend, UK |
| **FMO** |  |  |  |  |
| T1/ST2 | FITC | 1:200 | eBRG1 | eBioscience, US |

Lineage markers were placed on PE-Cy7 and APC-Cy7 which were used as dump channels. Leucocyte populations and single cells were identified using forward and side scatter characteristics and non-viable cells (7AAD^+^) were removed from further analysis. Lineage marker negative cells which were CD25^+^CD127^+^CD90.2^+^T1/ST2^+^ were regarded as ILC2 cells (as described by Halim and Takei, 2014).

**Histological preparation and analysis of lung tissue:**

*Histology:*

The left lung lobe was inflation-fixed with 4% paraformaldehyde. Single blocks were dehydrated through ascending grades of industrial methylated spirit (IMS), cleared with xylene and embedded in paraffin wax. Sections were cut at approximately 4µm, dried overnight, dewaxed in xylene, washed in IMS followed by running tap water and stained with Alcian Blue/Periodic Acid Schiff (AB/PAS). The slides were assessed using an Olympus BX41 microscope, scanned on an Aperio scanner and viewed and photographed using a Spectrum digital imaging system (Aperio).

*Immunohistochemistry:*

Paraformaldehyde fixed, paraffin-embedded sections were cut at approximately 4µm, air dried overnight and placed in an incubator, at 60ᵒ for 10 minutes. Sections were dewaxed in xylene, washed in IMS followed by running tap water. Antigen retrieval was performed with EDTA in a microwave for 10 minutes and cooled for 20 minutes. After washing in running tap water and Tris buffered saline (TBS) a REAL peroxidase block (Dako, S2023) was applied for 15 minutes. Following a TBS wash, a mouse on mouse block (ab12755, Abcam) was used for 30 minutes. After another TBS wash the primary antibody (anti-IL33 [Nessy-1], mouse monoclonal, protein concentration: 1000µg/ml, ab54385, Abcam) was applied for 1 hour. Extra sections were used as isotype controls and incubated with the same concentration of mouse IgG1 (Dako). The secondary antibody (anti-mouse HRP polymer, ab127055, Abcam) was applied for 30 minutes and following a TBS wash the reaction was visualised using DAB (Dako, 3468) and counterstained with haematoxylin. The sections were dehydrated in 2x2 minutes of IMS, cleared using 2x2 minutes in xylene and mounted in VWR Merck Entellan. The slides were assessed using an Olympus BX41 microscope, scanned on an Aperio scanner and viewed and photographed using a Spectrum digital imaging system (Aperio).

*Histological scoring:*

Goblet cell metaplasia:

Using AB/PAS staining, the number of goblet cells in the proximal airways was graded using a 3-point scale: +: minimal numbers; ++: slight increase in numbers; +++ moderate increase in numbers.

IL-33 expression:

Immunoreactivity (IR) in randomised slides sets was graded based on a 4-point scale: +=minimal; ++slight; +++moderate; ++++marked.

Supplementary Figure E1:

Supplementary Figure E1 legend:

HDM re-challenge initiated a complex lymphocyte response in the BAL and lungs. Most cell types in BAL peaked at day 7 post HDM re-challenge, but significant numbers were present at day 3. In general, significant numbers of CD4^+^ cells were present in lung tissue at day 3 in mice re-challenged to HDM compared to saline controls. However, there did not appear to be any difference between saline and HDM re-challenged mice in terms of lung CD8^+^ lymphocytes throughout the time-course investigated. NK cells in the lungs of mice re-challenged with HDM were significantly different to saline challenged animals only at the day 7 time-point post HDM re-challenge. (n=4 per time-point for saline controls and 6 for HDM re-challenged group). *p<0.05, **p<0.01, ***p<0.001, compared to saline controls.

Supplementary Figure E2:

Supplementary Figure E2 legend:

Effect of PI-3065, montelukast and anti-IL-5 antibody on numbers of lung resident Siglec-F^int^ eosinophils. Samples analysed at 24 hours post HDM re-challenge. Treatments did not significantly alter the numbers of lung resident eosinophils compared to their respective control groups (n=3-6 per group for saline controls and 6-11 for HDM re-challenged groups). A one-way analysis of variance (ANOVA) with Dunnets P value adjustment was used to analyse the data for significance.

Supplementary Figure E3: Pharmacokinetic data


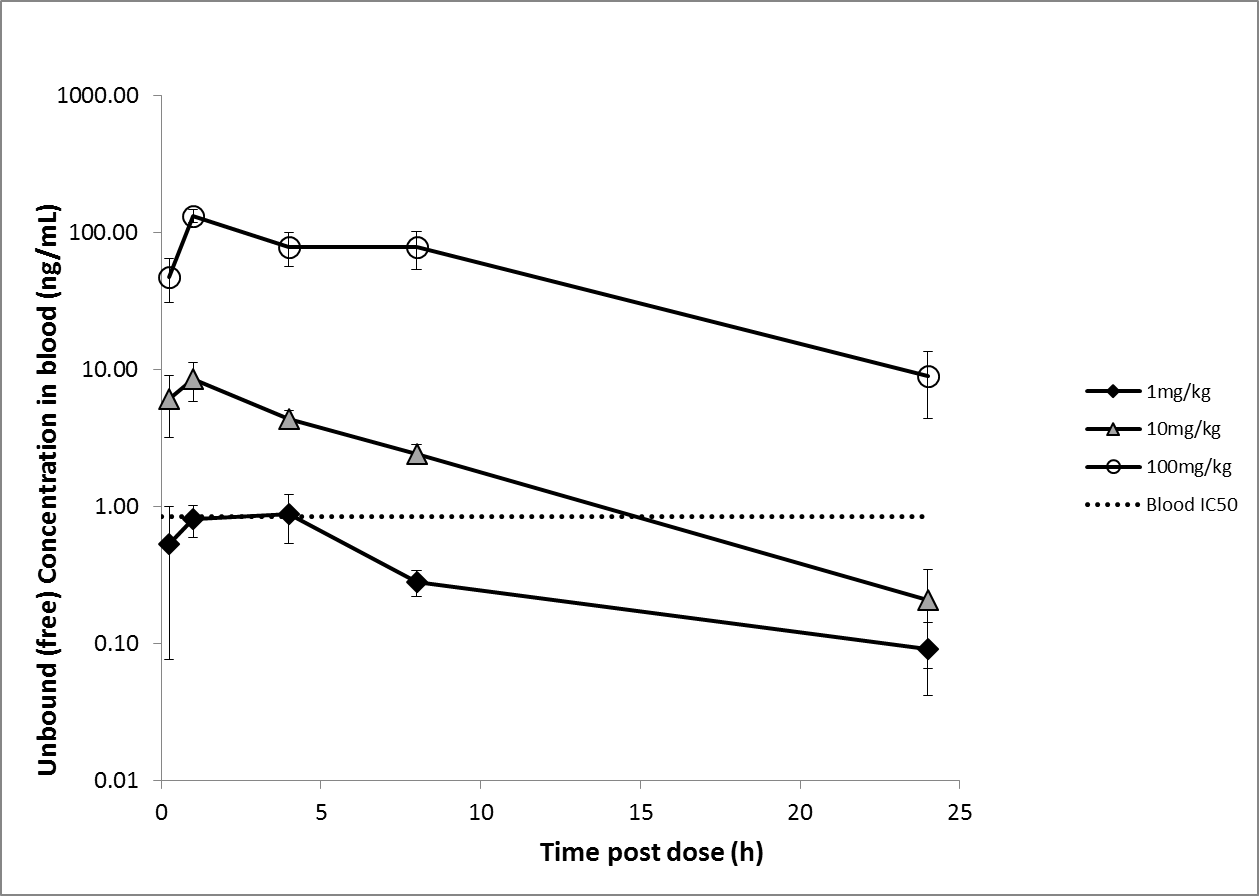


Supplementary Figure E3 legend:

Mean unbound concentration versus time profiles for PI-3065 in blood on day 1 following a single oral administration to female BALB/c mice at 1mg/kg, 10mg/kg and 100mg/kg, error bars represent the standard deviation of the mean (n=6). Blood samples were collected as live bleeds in a composite design. Terminal bleeds generated on day 7 (not shown) were in keeping with day 1 data suggesting no evidence for accumulation across the dosing period. IC50 values were generated using whole blood from *in vivo* study matched mice (female BALB/c mice treated with HDM for 3 weeks), values represent unbound concentration associated with 50% inhibition of HDM induced IL-4 release from mouse whole blood. Doses were selected to achieve concentrations at Cmax equivalent to IC50 (1mg/kg), equivalent to 10x IC50 and above IC50 for ~15hrs (10mg/kg) and above 10x IC50 for the dosing period (100mg/kg).
